# Hybrid Phase ordering with Automatic Window Selection (HybridPAWS) improves respiratory-navigator efficiency during 3D late-gadolinium enhancement CMR in patients with chronic heart failure and irregular respiratory pattern

**DOI:** 10.1186/1532-429X-14-S1-P256

**Published:** 2012-02-01

**Authors:** Zhong Chen, Christoph Kolbitsch, Jouke Smink, James Harrison, Valentina O Puntmann, Eike Nagel, Reza Razavi, Aldo Rinaldi, Tobias Schaeffter

**Affiliations:** 1 Imaging Sciences & Biomedical Engineering, Kings College London, London, UK

## Summary

Hybrid Phase ordering with Automatic Window Selection (HybridPAWS) improves respiratory-navigator efficiency during 3D late-gadolinium enhancement CMR in patients with chronic heart failure and irregular respiratory pattern. When there is a low gating efficiency (<40%) observed with standard respiratory-gated method, HybridPAWS technique resulted in a significantly greater improvement in navigator efficiency.

## Background

**Figure 1:**
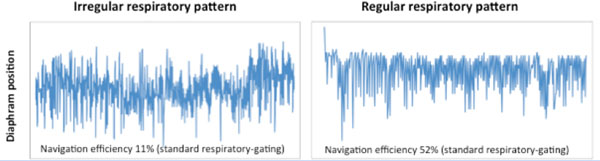
Example of irregular and regular breathing pattern detected by respiratory navigator windowed on the right hemi-diaphragm

Three-dimensional (3D) inversion recovery cardiac magnetic resonance (CMR) imaging is beginning to be utilised to characterise areas of late gadolinium enhancement (LGE) with high spatial resolution. This is important for tissue heterogeneity assessment in heart failure patients. The 3D CMR data are acquired during free breathing with the application of a respiratory navigator. However, a major challenge of its wider clinical application is the prolonged acquisition time in patients with irregular respiratory breathing pattern (Figure 1). The increased scan time can accentuate the effect of contrast kinetics on the quality of LGE-CMR images. In order to improve respiratory gating efficiency, we have implemented interleaved Radial Phase Encoding-Phase ordering with Automatic Window Selection for a Cartesian sampling scheme (HybridPAWS). With this HybridPAWS method, fast free-breathing 3D high-resolution LGE CMR data can be acquired in patients with irregular breathing pattern.

## Methods

Twenty-one patients underwent CMR imaging on a 1.5T CMR scanner (Philips medical System, Best, The Netherlands). HybridPAWS was implemented for respiratory-gated 3D inversion-recovery whole heart CMR data acquisition 25 minutes post contrast (gadolinium-DTPA, 0.2mmol/kg). Typical imaging parameters were FOV 300x300x100mm; TR/TE 5.7/2.7ms; flip angle 25°; acquired voxel size 1.3x1.3x2.6mm; trigger delay mid-diastole. The imaging time with the above resolution was typically 3.8 minutes assuming a navigator efficiency of 100% and heart rate of 60 beats-per-minute. The navigator efficiency was determined during the 3D inversion-recovery high-resolution CMR image acquisition with both the HybridPAWS and a standard respiratory-gated Cartesian method.

## Results

Eighteen patients had impaired left ventricular ejection function (LVEF) with a mean of 31% +/-11; NYHA heart failure symptom class 2-4. The other three patients had normal LVEF. The respiratory navigator efficiency was 45 +/- 12% with HybridPAWS and 36 +/- 16% with standard respiratory-gated Cartesian method; p<0.05. There was a mean 40 +/- 53% improvement in respiratory gating efficiency with HybridPAWS overall. When there is a low gating efficiency (<40%) observed with standard respiratory-gated method, HybridPAWS technique resulted in a significantly greater improvement in navigator efficiency (68 +/- 60% vs. 14 +/- 30%; p<0.05).

## Conclusions

Respiratory gating with HybridPAWS implementation in free-breathing 3D inversion-recovery whole heart scan results in a significant improvement in navigator efficiency whilst providing high spatial resolution. The greatest improvement with HypbridPAWS is observed in patients with irregular breathing pattern. This has a potential impact in reducing the effects of contrast kinetics on LGE-CMR image quality.

## Funding

N/A

**Figure 2:**
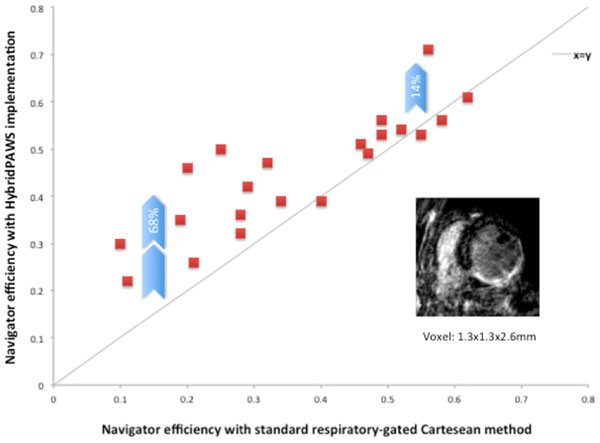
Respiratory gating efficiency with HybridPAWS vs. standard respiratory-gated Cartesian method in 3D high-resolution LGE-CMR

